# A role for auto-immunity in chronic rhinosinusitis? Lessons learned from sub-epidermal bullous disorders of the skin

**DOI:** 10.1186/s13223-016-0141-1

**Published:** 2016-08-05

**Authors:** Philippe Lefrançois, Hugo Chapdelaine, Benoît Côté, Martin Desrosiers

**Affiliations:** 1 Faculté de Médecine, Université de Montréal, Montreal, QC Canada; 2 Centre Hospitalier de l’Université de Montréal (CHUM), Université de Montréal, Montreal, QC Canada; 3 Centre de Recherche du Centre Hospitalier de l’Université de Montréal (CRCHUM), Université de Montréal, Montreal, QC Canada

**Keywords:** Chronic rhinosinusitis, Auto-immunity, IgE, Blistering skin disorders, Bullous pemphigoid

## Abstract

Chronic rhinosinusitis (CRS) is a frequent chronic condition, which has origins in complex interactions between genetic, immunological and microbial factors. The role of auto-immunity in CRS remains unclear, although recent studies have started to emerge in CRS patient refractory to maximal medical management. We discuss the possible auto-immunity link between CRS and other skin diseases, in particular acquired bullous dermatoses, and review the current evidence. We raise additional considerations for auto-immunity from both research and clinical standpoints.

## Background

Chronic rhinosinusitis (CRS) is a common condition affecting ~13 % of the US adult population, a prevalence higher than that of diabetes [1]. The productivity loss accounting from CRS amounts to $13 billion USD [2], with total disease burden estimated around ~$60 billion USD [3]. Of particular interest are patients with refractory CRS, for whom maximal medical therapy provides at most marginal benefits. Refractory CRS patients are currently managed with multiple sinus surgeries, yet they still experience recurrent exacerbations requiring antibiotics.

**Fig. 1:**
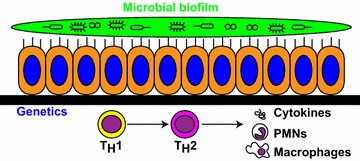
Chronic rhinosinusitis (CRS) as a multifactorial disease. Genetic factors affect the mucosal epithelium barrier, both structurally and functionally. Immune factors induce a strong T_H_2 bias, which leads to cytokine production and impaired innate inflammatory response. Microbial factors promote a dysbiotic flora and biofilm formation. *PMNs* polymorphonuclear leukocytes

The etiology of CRS is believed to be multifactorial **(**Fig. 1**)** [4]. Genetic predispositions most likely affect the role of the mucosal epithelium as a barrier, both structurally and functionally [4–7]. Immunity defects have long been known to be implicated in CRS, in particular for patients resistant to treatment [8]. Evidence for this includes the frequent occurrence of CRS in patients with common variable humoral immunodeficiency [9]. Indirect support for implication of other immune deficiency states include a disequilibrium of T_H_1/T_H_2, with a skewing towards stronger T_H_2 responses [10], aberrant *TLR* function in innate immunity [11], and chronic low-grade inflammation resulting from a constant feed-forward loop of pro-inflammatory cytokines [12]. A role is emerging for the microbiome, particularly in a model governed by gene–environment interactions, which would have as permanent members dysbiotic bacteria that colonize the chronically-inflamed mucosa to keep the latter dysfunctional and incorrectly repaired [13]. This has opened the door to novel therapeutic approaches, such as topical therapy with probiotics [14], dietary modifications [13, 15] and use of antibiotics as immuno-modulators and microbiome-modifiers [16] are all currently being investigated in the clinical setting.

## Main text

### Auto-immunity in CRS

Conspicuously absent from these descriptions is the possibility of a role for auto-immunity in CRS pathogenesis. This is somewhat surprising as auto-immunity is a ubiquitous culprit in pathologies affecting every other organ, thus it is likely that a similar phenomenon also occurs in CRS. Here we explore the auto-immune avenue, with a focus on comparison with chronic auto-immune conditions compromising the skin epidermal barrier.

### Evidence from immune complex disorders

The deposition of auto-immune complexes in the sinus mucosa disrupting the sinus mucosal surface is already demonstrated in vasculitides [17]. Vasculitides which can present with sinusitis symptoms include granulomatosis with polyangiitis (GPA; formerly known as Wegener’s granulomatosis) [18] and eosinophilic granulomatosis with polyangiitis (EGPA; Churg-Strauss Syndrome) [19]. Immune complexes formed by anti-neutrophil cytoplasmic auto-IgG antibodies (ANCA IgG) promote granuloma formation in the epithelium, effectively causing a chronically-inflamed permeable barrier [20, 21]. Although this constitutes an interesting autoimmune hypothesis for CRS, the severity of disease seen in these patients and the low prevalence of these disorders are not sufficient to account for the frequency of CRS. As an additional consideration, these two disorders differ markedly in their immunologic profile: GPA predominantly displays a T_H_1 response while immunity in EGPA is shifted towards T_H_2 cytokines, with both disorders showing T_H_17 profiles [22].

Given the pathophysiological differences between GPA and CRS, we postulate that other manifestations of auto-immunity are likely present in CRS. Since sinus mucosa represents an ectodermal barrier between internal microenvironments and external aggressors, CRS may share features with other barrier disorders, such as the skin.

### Auto-immunity and blistering skin disorders

Auto-immune sub-epidermal blistering diseases of the skin epithelium include various acquired bullous disorders such as bullous pemphigoid, cicatricial pemphigoid, pemphigoid gestationis and epidermolysis bullosa acquisita [23]. Auto-antibodies are classically directed against components of the hemidesmosome-anchoring filament complexes or anchoring fibrils, which link the basement membrane to the basal keratinocyte layer via cytoskeletal elements [24]. These targeted proteins at the dermal–epidermal junction include laminins, collagens, integrins and other cell adhesion molecules [24].

Cicatricial, or mucous membrane, pemphigoid is a chronic auto-immune blistering disease that preferentially affects the oral and ocular mucosae, but can also affect the nasal mucosa and the genitalia [23]. Cicatricial pemphigoid shows the highest heterogeneity among auto-immune bullous conditions, with the identification of auto IgA and/or IgG targeting the α6 integrin subunit, β4 integrin subunit, laminin 5 and/or BP180/collagen XVII α1 [25]. Pemphigoid, or herpes gestationis is a bullous eruption of the third trimester and immediate post-partum that extends from the umbilicus in a centrifugal direction [26]. In pemphigoid gestationis, a linear C3 deposition at the dermo-epidermal junctional is nearly always found, but auto IgG against BP180/collagen XVII and, less commonly, BP230 can be detected in a third of affected pregnancies [24, 26]. Similarly to other dermatoses of pregnancy, pemphigoid gestationis displays an immune imbalance towards a stronger T_H_2 response with concomitant depressed T_H_1 function [27]. Epidermolysis bullosa acquisita is characterized by recurring blisters on skin and mucosae arising from IgG auto-immunity against collagen VII [28, 29].

### Insights from bullous pemphigoid

Studies from bullous pemphigoid (BP), a more common blistering disease of the elderly, have shed insights into the link between a dysbiotic flora and auto-immunity [30]. Auto IgG, and to a much lesser extent, auto IgA, against BP180/collagen XVII and BP230, a transmembrane protein of the hemidesmosal plaque, have been implicated in the pathogenesis of BP for many years [31, 32]. Recently, auto IgE antibodies have been consistently isolated from a subset of patients with BP, both at their dermal-epidermal junction [33, 34] and in their sera [35, 36]. This led to the successful use of omalizumab, a humanized monoclonal that binds and inactivates free and membrane-bound IgE, to manage patients with standard treatment-refractory BP and elevated IgE titers [37, 38].

**Fig. 2:**
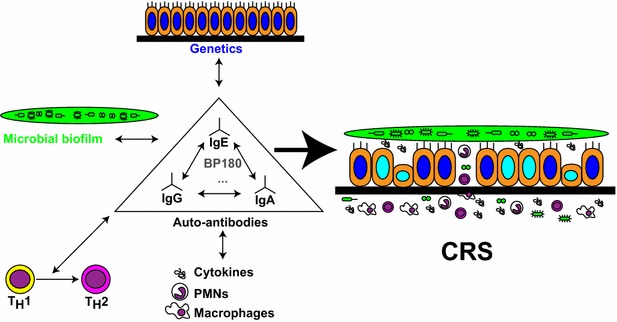
Probable interplay between auto-antibodies and known contributing factors leading to CRS. Auto-antibodies, whether IgG, IgE or IgA, influence microbial, genetic and immune factors. The latter would probably exert a feed-forward retroaction, leading to increased levels of auto-antibodies. As shown in other auto-immune disorders, different classes of auto-Ig have synergistic effect on other classes. An auto-antibody targeting BP180 could be implicated in CRS pathogenesis. Others are likely to be discovered. Together, these factors maintain the chronic inflammatory state of CRS, with its altered microbial flora, dysfunctional epithelium, impaired barrier function and self-propagating inflammation

How does the presence of auto IgE in BP relate to CRS? First, IgE dysfunction and impairment of mast cell regulation and response are involved in various type I hypersensitivity reactions, such as asthma, allergic rhinitis, atopic dermatitis, and urticaria [39, 40]. Of note, CRS severity is strongly associated with the presence of asthma, nasal polyposis, aspirin allergy and atopy in general [41]. Serum IgE levels are elevated by 9- to 20-fold in CRS patients (without or with nasal polyposis) [42]. Second, the use of omalizumab in refractory CRS patients with nasal polyposis has also improved symptomatology, significantly [43, 44] in some studies and non-significantly [45] in others. In the latest randomized clinical trial, this significant effect was present in patients with both allergic and non-allergic conditions [44], suggesting that the mechanisms of action is not simply limited to Type I hypersensitivity. Third, auto IgE antibodies can act in synergy with other auto-antibodies to mediate auto-immunity. In patients suffering from systemic lupus erythematous, auto-IgE antibodies, and the deposition of immune complexes containing auto-IgE, potentiate the effect of the more abundant auto-IgG antibodies (~20 fold difference), leading to higher activation of innate inflammatory components, thus predicting a worse disease course [46]. Similarly, in CRS, auto-IgE antibodies could mediate the interplay between mucosal innate defenses, acquired auto-immunity, and microbial agents, despite their relative paucity (Fig. 2).

### BP180 auto-antibodies in CRS

The discovery of autoantibodies in CRS patients is emerging. Anti-nuclear IgG and IgA autoantibody titers are elevated in CRS patients with nasal polyposis [47]. Recently, the discovery of elevated circulating levels of auto IgG against BP180/collagen XVII in CRS patients vs. healthy patients bring the link with bullous pemphigoid back into the spotlight [48]. However, deposition of these autoantibodies at the dermal-epidermal junction has been absent from the nasal mucosa of CRS patients, in stark contrast with BP in which it is pathogenic [48]. Nevertheless, auto-immunity appears to play an important role in CRS, and similarities to BP and other acquired skin bullous dermatoses are numerous.

### Genetics, CRS and skin bullous conditions

Other links to epithelial blistering disorders are suggested by genome-wide association studies (GWAS) of refractory CRS, in which several of the most common gene polymorphisms in CRS patients code for basement membrane and extra-cellular matrix proteins, including the laminins [6, 49]. Other polymorphisms associated with CRS are found in collagen- and keratin-interacting genes [49]; the complete absence of these genes and related genes as well as aberrant gene expression can lead to some of the rare hereditary epidermolysis bullosa genodermatoses [50]. Taken together, these findings suggest that interference with epithelial structure leading to epithelial blistering disorders in skin may mimic changes seen in CRS.

### CRS similarities to other skin conditions

In addition to skin bullous diseases, other skin conditions may yield insight into the pathophysiology of CRS. In chronic urticaria, dysregulated cellular function and autoantibodies targeting directly IgE or their receptor on mast cells lead to the degranulation of mast cells and subsequent release of pre-formed pro-inflammatory cytokines [51]. In CRS with nasal polyposis, mast cells in the epithelium and glandular structures are increased in number and are producing abnormally-high quantities of proteases, thus contributing to the chronic inflammation and lack of complete repair [52]. Circulating auto-IgE antibodies have also been detected in chronic urticaria patients [53].

## Conclusion

CRS likely arises from the interaction of genetic, immunological and microbial factors. This concept may evolve to incorporate a contribution from auto immune disorders. While experience with autoimmunity in CRS is still very limited, insights may be drawn from experience with skin epidermal disorders. Notably, in skin, auto-immunity in epidermis-basement membrane unit renders the epidermis dysfunctional and more permeable to surface pathogens, which may penetrate into the dermis, enabling the colonization by a dysbiotic flora that maintains a chronic inflammation state. This contributes to the secondary mucosal changes seen in skin disorders. These concepts resonate well with the disease process observed in CRS. The role of auto-immunity in CRS, especially in refractory cases, appears compelling. Given the aforementioned similarities between sub-epidermal bullous dermatoses, focusing on the basal keratinocyte–hemidesmosome–cytoskeleton–basal membrane interface might reveal additional mechanistic information. Additionally, it is important to recognize that not all autoimmune actors are IgG auto-antibodies, and that IgA and IgE auto-antibodies may be more common than expected. Assessment for these should also be performed. As previously mentioned in the case of anti-BP180 in CRS [48], it is important to distinguish circulating auto-antibodies from those bound to the epithelium as these are probably significant for the maintenance of the disease. Nevertheless, dosing circulating auto-antibodies may serve as a proxy for detecting auto-immunity. As in BP and chronic urticaria, refractory CRS might also feature auto-IgE antibodies.

Suggestions for further studies include (1) verifying the presence of other circulating auto-antibodies targeting the dermal–epidermal junction and the hemidesmosome plaque in CRS patients, and (2) determining the presence of auto-antibodies on sinus biopsies/tissues and nasal polyps from CRS patients using dermato-histopathological techniques.

On the clinical side, implications of these findings are twofold. First, investigation of CRS should include the possibility of auto-immunity. Second, clinicians may have to consider biological and targeted therapies aimed at decreasing auto-immunity in refractory CRS patients. Experience with those treatment regimens from diseases involving other body systems should come in handy for CRS. However, therapeutic agents used in other disorders will require prospective assessment prior to clinical deployment.
